# TMG-123, a novel glucokinase activator, exerts durable effects on hyperglycemia without increasing triglyceride in diabetic animal models

**DOI:** 10.1371/journal.pone.0172252

**Published:** 2017-02-16

**Authors:** Yoshinori Tsumura, Yu Tsushima, Azusa Tamura, Makiko Hasebe, Masanobu Kanou, Hirotsugu Kato, Tsunefumi Kobayashi

**Affiliations:** Pharmaceutical Development Research Laboratories, Teijin Pharma Limited, Hino, Tokyo, Japan; Stellenbosch University, SOUTH AFRICA

## Abstract

Glucokinase (GK) plays a critical role for maintaining glucose homeostasis with regulating glucose uptake in liver and insulin secretion in pancreas. GK activators have been reported to decrease blood glucose levels in patients with type 2 diabetes mellitus. However, clinical development of GK activators has failed due to the loss of glucose-lowering effects and increased plasma triglyceride levels after chronic treatment. Here, we generated a novel GK activator, TMG-123, examined its *in vitro* and *in vivo* pharmacological characteristics, and evaluated its risks of aforementioned clinical issues. TMG-123 selectively activated GK enzyme activity without increasing Vmax. TMG-123 improved glucose tolerance without increasing plasma insulin levels in both insulin-deficient (Goto-Kakizaki rats) and insulin-resistant (db/db mice) models. The beneficial effect on glucose tolerance was greater than results observed with clinically available antidiabetic drugs such as metformin and glibenclamide in Zucker Diabetic Fatty rats. TMG-123 also improved glucose tolerance in combination with metformin. After 4 weeks of administration, TMG-123 reduced the Hemoglobin A1c levels without affecting liver and plasma triglyceride levels in Goto-Kakizaki rats and Diet-Induced Obesity mice. Moreover, TMG-123 sustained its effect on Hemoglobin A1c levels even after 24 weeks of administration without affecting triglycerides. Taken together, these data indicate that TMG-123 exerts glucose-lowering effects in both insulin-deficient and -resistant diabetes, and sustains reduced Hemoglobin A1c levels without affecting hepatic and plasma triglycerides even after chronic treatment. Therefore, TMG-123 is expected to be an antidiabetic drug that overcomes the concerns previously reported with other GK activators.

## Introduction

Type 2 diabetes mellitus (T2DM) is a metabolic disease characterized by chronic hyperglycemia primarily resulting from defects in insulin secretion, insulin efficacy, or both [1]. For managing hyperglycemia to prevent microvascular and macrovascular complications, drug therapy is recommended in addition to healthy diet and exercise therapies [2]. Current major drug therapy for T2DM can be categorized into six classes (Biguanides, Sulfonylureas, Thiazolidinediones, DPP-4 inhibitors, SGLT-2 inhibitors, and GLP-1 receptor agonists) in addition to insulin analogs. Those drugs exert various physiological actions; increase of insulin secretion or insulin sensitivity, decrease of hepatic glucose production, blocks glucose reabsorption by kidney, and so on [3]. Although many classes of antidiabetic drugs are currently available, there remains a continued need for novel drugs to achieve treatment goals. Therefore, several drugs with a novel mechanism of action are now under development [4].

Glucokinase (GK), also known as hexokinase (HK) type IV, is one of the four HK family members. It catalyzes the first reaction in glucose metabolism, phosphorylating glucose to glucose-6-phosphate [5, 6]. Affinity of GK for glucose is low compared to other HKs, and Km values were around the physiological blood glucose levels (5–7 mM) [5, 6]. Expression of GK is limited to the major organs (pancreas, liver, brain, and gastrointestinal tract) [7]. GK particularly regulates the secretion of insulin in response to rising levels of blood glucose [8] and the rate of hepatic glucose uptake and glycogen synthesis in liver [9]. These characteristics allow GK to act as a glucose sensor and to regulate whole body glucose homeostasis. Therefore, activation of GK is expected to be successful and novel therapeutic strategy for T2DM [10].

To date, several GK activators have been reported to decrease Hemoglobin A1c (HbA1c) levels in patients with T2DM [11–15]. In spite of their positive effects, GK activator treatment has also been associated with unfavorable effects. Certain clinical trials have revealed the loss of efficacy over time and an increase in circulating triglyceride (TG) levels. In preclinical studies using rodent models, several GK activators have been reported to exert glucose-lowering effects as well as increasing plasma TG [16, 17]. Accordingly, it is important to evaluate the pharmacological characteristics in experimental studies before conducting clinical trials.

Here, we generated a novel GK activator TMG-123, which is currently under clinical development. In this preclinical study, we examined the *in vitro* and *in vivo* pharmacological characteristics of TMG-123 as an antidiabetic drug and evaluated the risks of loss of efficacy and increased TG levels.

## Materials and methods

### Materials

Recombinant human, rat, and mouse GK of liver and pancreas isoforms were produced by Teijin Pharma Limited (Tokyo, Japan). Human recombinant HK I, II, and III were purchased from ATgen (Seongnam-si, Korea). 2-[1,2-^3^H(N)]Deoxyglucose was obtaind from PerkinElmer (Boston, MA, USA). MIN6 cells [18] were obtained from Prof. Miyazaki (Graduate School of Medicine/Division of Medicine, Osaka University, Osaka, Japan). TMG-123 was synthesized by Teijin Pharma Limited. Metformin and Glibenclamide were obtained from Wako Pure Chemical Industries, Ltd. (Osaka, Japan).

### Animals

Male Sprague-Dawley rats, male ZDF-Lepr^fa^/CrlCrlj(genotype: *Lepr*^*fa*^/*Lepr*^*fa*^) (ZDF) rats, male BKS.Cg-*Dock7*^*m*^ +/+ *Lepr*^*db*^/J(genotype: + *Lepr*^*db*^/ + *Lepr*^*db*^) (db/db) mice, male C57BL/6J mice, and male C57BL/6J-DIO mice were obtained from Charles River Laboratories Japan, Inc. (Kanagawa, Japan). Male Goto-Kakizaki rats and Wistar rats were obtained from Japan SLC, Inc. (Shizuoka, Japan). All animals were housed under a 12 h light-dark cycle and were fed ad libitum a CE-2 (CLEA Japan, Inc., Tokyo, Japan), high fat (60Kcal% fat) diet (D12492, RESEARCH DIETS, Inc., New Brunswick, NJ, USA), or Purina 5008 (Purina Mills, St. Louis, MO, USA); CE-2 for db/db mice, C57BL/6J mice, Sprague-Dawley rats, Goto-Kakizaki rats, and Wistar rats, high fat diet for C57BL/6J-DIO mice, and Purina 5008 for ZDF rats. All experimental procedures except oral glucose tolerance test (OGTT) in db/db mice were approved by the Animal Care and Use Committee of Teijin Institute for Bio-Medical Research and OGTT in db/db mice was approved by the Animal Ethical Committee of Kyorin Pharmaceutical Co.,Ltd (Tochigi, Japan). All efforts were made to minimize suffering. These laboratory animal facilities were accredited by the Center for Accreditation of Laboratory Animal Care and Use, Japan Health Sciences Foundation (Permit Number: 13–066, 15–051).

### Enzyme activity assays

GK and HK activity assays were performed according to a previous report [19]. In these assays, GK or HK converts glucose to glucose-6-phosphate, which is converted to 6-phosphoglucono-D-lactone by glucose-6-phosphate dehydrogenase along with the conversion of NAD^+^ to NADH. The change per unit time in NADH (absorbance at 340 nm) produced in the enzyme reaction was calculated, and enzyme activity was obtained by adjusting this value with the NADH absorbance coefficient (6.22 mM^-1^ cm^-1^) and each enzyme concentration. All enzyme assays were performed in the presence of 25 mM HEPES, pH 7.1, 25 mM KCl, 2 mM MgCl_2_, 1 mM ATP, 1 mM NAD^+^, 1 mM DTT, 5 units/mL glucose-6-phosphate dehydrogenase, 0.1% BSA, and 5% DMSO.

### *In vitro* glucose uptake assay in primary rat hepatocytes

Primary hepatocytes were isolated by collagenase perfusion of liver from 12-week-old male Sprague-Dawley rats as previously described [20], and plated on collagen-coated 24 well plates at a density of 4.0×10^4^ cells/well in DMEM containing 5 mM glucose, 10% FBS, and 100 unit/mL penicillin/streptomycin. The cells were allowed to attach to the plate for 4 hours, and the media was then changed to DMEM containing 1 mM glucose, 0.2% BSA (fatty acid free), 10 nM dexamethasone and 1 nM insulin. After 14–15 hours, the media was changed again to DMEM containing 4 mM glucose, 0.2% BSA (fatty acid free), and 1 μCi/mL 2-[1,2-^3^H(N)]deoxyglucose, and the cells were incubated for 8 hours in the presence of TMG-123. The medium was subsequently removed and the cells were washed three times with PBS. The cells were then lysed with 30% potassium hydroxide, the lysates were mixed with Ultima Gold^TM^, and radio activity was measured using a liquid scintillation counter (LS-6500, Beckman Coulter, Inc., Brea, CA, USA). The glucose analog 2-deoxyglucose was used to evaluate glucose uptake by measuring the hepatocyte uptake of 2-[1,2-^3^H(N)]deoxyglucose.

### *In vitro* insulin secretion assay in MIN6 cells

MIN6 cells were routinely maintained in DMEM containing 25 mM glucose and 15% FBS at 37°C with 5% CO_2_. For the assay, MIN6 cells were plated at a density of 2×10^5^ cells/well into 24 well plates. After one day, the cells were washed with PBS, and incubated for one hour in Krebs-Ringer bicarbonate HEPES buffer containing 0.1% BSA (0.1% BSA-KRBH). Thereafter, the media was replaced to 0.1% BSA-KRBH containing 4 mM glucose and various concentrations of TMG-123. After incubation for one hour, insulin concentrations in the culture media were determined using a Morinaga Insulin Determination Kit (Morinaga Institue of Biological Science, Inc., Kanagawa, Japan).

### OGTT following single administration of agents

Goto-Kakizaki rats at 10 weeks of age, db/db mice at 6 weeks of age, and ZDF rats at 6 weeks of age were fasted for 16–20 hours prior to OGTT. Animals were orally administered TMG-123 (1–30 mg/kg), metformin (30–300 mg/kg), glibenclamide (1–10 mg/kg), or vehicle (Gelucire/Polyethylene Glycol 400 (PEG400) = 3/2). Thirty or sixty minutes later, the animals were also orally administered glucose (rats; 2 g/kg, mice; 5 g/kg). Blood was sampled at -30 or -60 minutes prior to drug administration (TMG-123, metformin, glibenclamide, or vehicle), at time 0 minutes (prior to glucose administration), and at 0.25, 0.5, 1, 2, and 4 hours after glucose administration. Plasma glucose was measured using a Glucose CII-Test Wako (Wako Pure Chemical Industries, Ltd.), and plasma insulin was measured by High-sensitive Measurement Kit for Insulin (Morinaga Institute of Biological Science, Inc.).

### Sub-chronic and chronic treatment study

Goto-Kakizaki rats at 6 weeks of age were administered TMG-123 (12.5–50 mg/kg) or vehicle (Gelucire/PEG400 = 3/2) and Wistar rats at 6 weeks of age were administered vehicle once daily for 4weeks. After the administration, blood was collected for the measurement of HbA1c levels using a DCA 2000 system (Siemens, Munich, Germany). The rats were then sacrificed under ad libitum feeding conditions and blood and liver were collected. Plasma TG levels were measured using an Autosera^®^ S TG-N (SEKISUI MEDICAL Co., Ltd., Tokyo, Japan). TG in liver was extracted with 2-propanol and measured using a Wako L-Type TG H kit (Wako Pure Chemical Industries, Ltd.).

C57BL/6J-DIO mice at 12 weeks of age were fed for 4 or 24 weeks with high fat diet containing TMG-123 (0.002–0.1%). C57BL/6J mice at 12 weeks of age were fed with CE-2 as a normal control. In the 4-week study, blood was collected for the measurement of HbA1c levels after 4 weeks of treatment. In the 24-week study, blood was sampled for the measurement of HbA1c levels and plasma insulin levels every 4 weeks. After 4 or 24 weeks of treatment, mice were sacrificed under ad libitum feeding condition and blood and liver were collected. Plasma and liver TG levels were evaluated in the same manner as with the Goto-Kakizaki rats.

### Statistical analysis

Statistical analysis was performed using SAS software version 8.2 or 9.2 (SAS Institute Inc., Cary, NC, USA). Data are expressed as mean + SEM in each figure. The statistical significance of differences was assessed using two-tailed Student’s t-test and the Aspin-Welch test for single comparisons, or one-way ANOVA followed by Dunnett’s test, Steel’s test or Tukey’s test for multiple comparisons, or the one-tailed Williams’ test or Shirley-Williams’ test for dose-dependent studies. Differences were considered significant when p values were < 0.025 in the Williams’ test and Shirley-Williams’ test or < 0.05 in all other tests. The concentration-response data were fitted, and EC_50_ values and Vmax were obtained using GraphPad PRISM software version 4.03, 5.02 or 6.07 (GraphPad, La Jolla, CA, USA).

## Results

### Characterization of TMG-123 in enzyme assays

**Fig 1:**
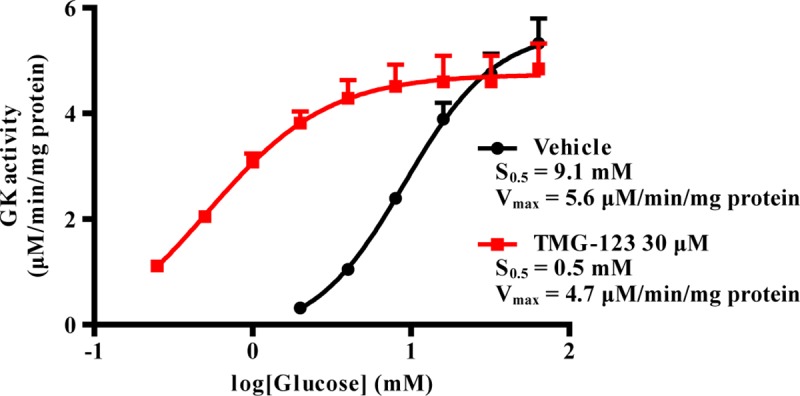
TMG-123 decreases S_0.5_ value of GK without increasing Vmax. Glucose concentration-versus-human liver GK activity relationships in the presence of 30 μM TMG-123 or vehicle alone (5% DMSO).

We performed enzymatic assays to evaluate the potency of TMG-123 for GK activation. It has been reported that different GK isoforms are produced by tissue-specific alternative RNA splicing in the liver and pancreas [21]. We examined the effects of TMG-123 on human liver and pancreas GK. EC_50_ values observed in the presence of 5 mM glucose for human liver and pancreas GK were 0.35 μM and 0.32 μM, respectively, suggesting that TMG-123 can equally activate both isoforms (Table 1). To determine the difference between species, we also examined the effect of TMG-123 on rat and mouse GK. EC_50_ values of rat liver and pancreas GK were 0.32 and 0.36 μM, and those of mouse were 0.38 and 0.32 μM, respectively (Table 1). These results indicate the absence of species differences in the effects of TMG-123. GK is one of the members of the HK family, which contains HKI, II and III [5]. To confirm the selectivity of TMG-123, we examined the effects of TMG-123 on HKI, II, and III. In the presence of 0.25 mM glucose, TMG-123 (1–100 μM) did not alter the activities of HKI, II, and III (Table 2), indicating that TMG-123 selectively activates GK over other HK family members. Next, to examine the effect of TMG-123 on the kinetic properties of GK, the activity was measured in the presence of various glucose concentrations. Human liver GK was glucose concentration-dependently activated in the absence of TMG-123, and the S_0.5_ and Vmax values were 9.1 mM and 5.6 μM/min/mg protein, respectively (Fig 1). TMG-123 increased the affinity of liver GK for glucose, and the S_0.5_ value was 0.5 mM in the presence of 30 μM TMG-123. Conversely, the Vmax value did not change, and in the presence of 30 μM TMG-123, the Vmax value was 4.7 μM/min/mg protein (Fig 1). Similar results were obtained with pancreas GK (S1 Fig). The results demonstrate that TMG-123 increases the affinity of GK to glucose without increasing Vmax.

**Table 1:** EC_50_ values for GK activation by TMG-123.

	EC_50_ values (μM)	
species	Liver GK	Pancreas GK
Human	0.35 ± 0.05	0.32 ± 0.04
Rat	0.32 ± 0.05	0.36 ± 0.04
Mouse	0.38 ± 0.04	0.32 ± 0.04

Data shown as mean ± SEM

**Table 2:** HK activations by various concentrations of TMG-123.

	HK activities (% of Control)		
TMG-123 (μM)	HK I	HK II	HK III
1	99 ± 4	104 ± 2	104 ± 2
10	99 ± 3	103 ± 3	102 ± 1
100	87 ± 7	102 ± 3	94 ± 4

Data shown as mean ± SEM

### Effects of TMG-123 on glucose uptake in hepatocytes and insulin secretion in β-cells

**Fig 2:**
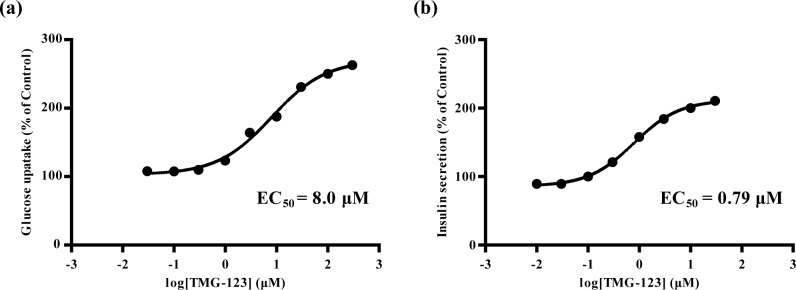
TMG-123 increases glucose uptake in rat primary hepatocytes and insulin secretion in MIN6 cells. (a) Glucose uptake in rat primary hepatocytes in the presence of 4 mM glucose is calculated by determining uptake of 2-[1,2-^3^H(N)]deoxyglucose after 8 hours of treatment with several concentrations of TMG-123. (b) Insulin secretion from MIN6 cells in the presence of 4 mM glucose after 1 hour of treatment with several concentrations of TMG-123.

We evaluated effects of TMG-123 on glucose uptake and insulin secretion in cell-based assays. To evaluate glucose uptake, we isolated primary hepatocytes from Sprague-Dawley rats and measured the amount of 2-[1,2-^3^H(N)]deoxyglucose uptake by hepatocytes. At 4 mM glucose, TMG-123 (30 nM-300 μM) increased glucose uptake in a concentration-dependent manner, with 300 μM TMG-123 increasing uptake 2.6 times more than control (Fig 2A). The EC_50_ value of TMG-123 was 8.0 μM. To evaluate insulin secretion, we cultured the pancreatic β-cell line MIN6 and measured the concentrations of insulin in the culture media to determine the amount of insulin secretion after treatment with TMG-123. At 4 mM glucose, TMG-123 (10 nM-30 μM) increased insulin secretion in a concentration-dependent manner, with 30 μM TMG-123 increasing secretion 2.1 times more than control (Fig 2B). The EC_50_ value of TMG-123 was 0.79 μM. Taken together, these results suggest that TMG-123 has the potential to enhance glucose uptake in hepatocytes and stimulate insulin secretion in pancreatic β-cells.

### Effects of TMG-123 on glucose tolerance in animal models of insulin-deficient and -resistant diabetes

**Fig 3:**
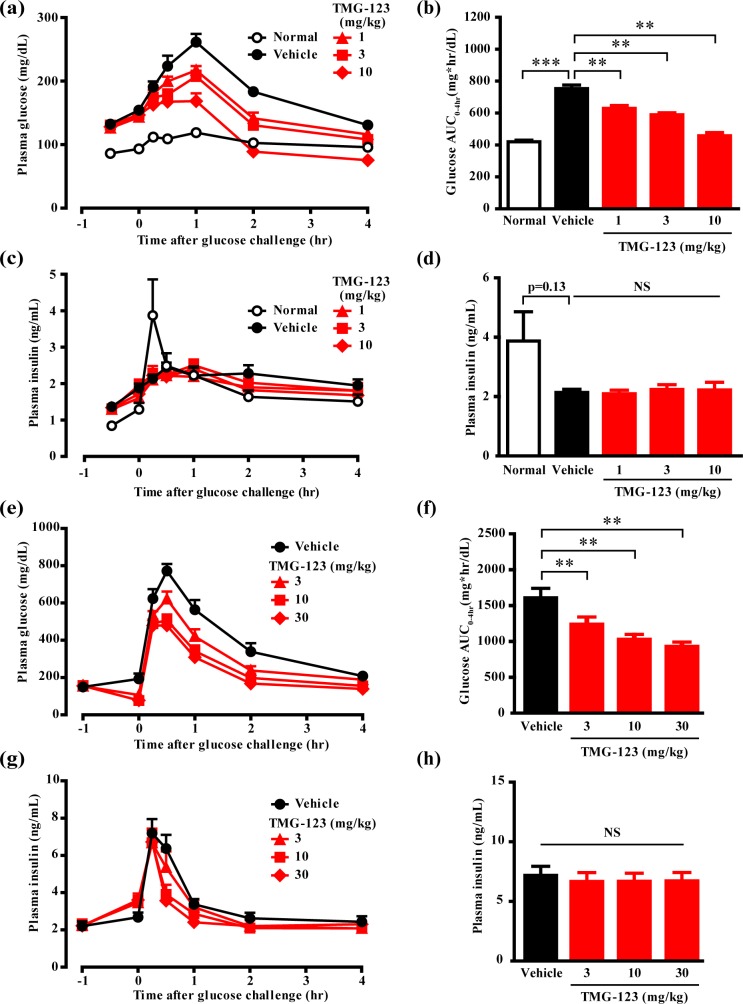
Administration of TMG-123 improves glucose tolerance without stimulating insulin secretion in animal models of insulin-deficient and -resistant diabetes. (a-d) OGTTs with Goto-Kakizaki rats and Wistar rats. TMG-123 (1–10 mg/kg) or vehicle (Gelucire/PEG400 = 3/2) were orally administered at -0.5 hr, and 2 g/kg of glucose was orally gavaged at 0 hr (n = 8, each). (a) Plasma glucose curves and (b) Glucose AUC_0-4hr_ following OGTTs in Goto-Kakizaki and Wistar rats. (c) Plasma insulin curves and (d) plasma insulin levels at 0.25 hr following OGTTs in Goto-Kakizaki and Wistar rats. (e-h) OGTTs in db/db mice. TMG-123 (3–30 mg/kg) or vehicle were orally administered at -1 hr and 5 g/kg glucose was orally gavaged at 0 hr (n = 8, each). (e) Plasma glucose curves and (f) Glucose AUC_0-4hr_ following OGTTs in db/db mice. (g) Plasma insulin curves and (h) plasma insulin levels at 0.25 hr following OGTTs in db/db mice. **p < 0.025, ***p < 0.001, NS = not significant.

To investigate the effects of TMG-123 on whole body glucose metabolism *in vivo*, we performed OGTT in animal models of T2DM. First, we evaluated the effect of TMG-123 in an insulin-deficient model, Goto-Kakizaki rats. Plasma glucose remained at higher levels in the vehicle-treated group (Goto-Kakizaki rats) compared to the normal group (Wistar rats), for 4 hours after glucose loading (Fig 3A). Moreover, plasma insulin levels increased in the normal group 0.25 hours after glucose loading, but not in the vehicle-treated group (Fig 3C). These results were consistent with a previous study that reported impaired glucose tolerance with insulin deficiency in Goto-Kakizaki rats [22]. Compared to the vehicle, TMG-123 (1–10 mg/kg) dose-dependently decreased plasma glucose levels, and significantly decreased the area under the curve for plasma glucose levels from glucose loading to 4 hours later (Glucose AUC_0-4hr_) (Fig 3B). Additionally, TMG-123 did not decrease plasma glucose below 70 mg/dL, even at the highest dose. Unexpectedly, the plasma insulin levels of the TMG-123-treated groups were not higher than those of the vehicle-treated group at 0.25 hours after glucose loading (Fig 3D). Next, we evaluated the effect of TMG-123 on glucose tolerance in insulin-resistance model (db/db mice). Administration of TMG-123 reduced glucose levels in the fasting state (Fig 3E). Glucose levels following the oral glucose challenge in the TMG-123-treated groups were also lower than in the vehicle-treated group, and glucose AUC_0-4hr_ significantly decreased in a dose-dependent manner (Fig 3E and 3F). At all doses of TMG-123, plasma glucose levels did not decrease below 70 mg/dL. Even in db/db mice which retained glucose concentration-dependent insulin secretion, TMG-123 did not affect plasma insulin levels throughout the observation period (Fig 3G) and at the peak of insulin secretion (Fig 3H). These results demonstrate that TMG-123 improves glucose tolerance in both insulin-deficient and -resistant diabetes models. Moreover, since TMG-123 did not increase plasma insulin levels, it is assumed that TMG-123 improves glucose tolerance not by the stimulating insulin secretion, but mainly by enhancing hepatic glucose uptake.

### Comparison of the effects of TMG-123 on glucose tolerance with those of other agents for diabetes

**Fig 4:**
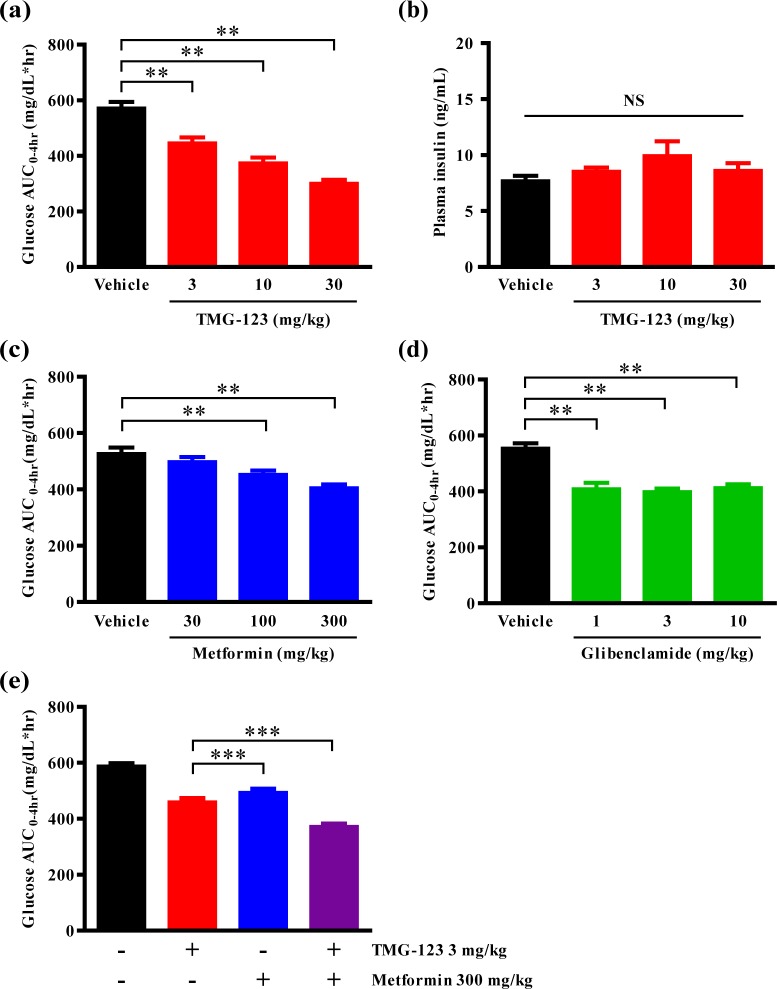
Administration of TMG-123 improves glucose tolerance more than metformin and glibenclamide, and exerts additional efficacy in combination with metformin. (a-e) OGTTs in ZDF rats. TMG-123 (3–30 mg/kg), metformin (30–300 mg/kg), glibenclamide (1–10 mg/kg), TMG-123 plus metformin or vehicle (Gelucire/PEG400 = 3/2) were orally administered at -0.5 hr and 2 g/kg of glucose was orally gavaged at 0 hr (n = 9–10). (a, c, d, e) Glucose AUC_0-4hr_ and (b) plasma insulin levels at 0.25 hr following OGTTs in ZDF rats. **p < 0.025, ***p < 0.001, NS = not significant.

To estimate the potency of TMG-123, we compared the effect of TMG-123 on glucose tolerance to those of metformin, one of Biguanides, and glibenclamide, one of Sulfonylureas, in another diabetic animal model (ZDF rats). We conducted the OGTT after the oral administration of TMG-123 (3–30 mg/kg), metformin (30–300 mg/kg), or glibenclamide (1–10 mg/kg), and evaluated plasma glucose levels until 4 hours after the glucose load. TMG-123 dose-dependently decreased glucose AUC_0-4hr_, with AUC_0-4hr_ at 30 mg/kg significantly reduced to 299.1 mg/dL*hr (Fig 4A). Similar to other diabetic animal models, TMG-123 did not increase the plasma insulin levels (Fig 4B). Metformin dose-dependently decreased glucose AUC_0-4hr_, with AUC_0-4hr_ at 300 mg/kg significantly reduced to 403.6 mg/dL*hr (Fig 4C). Glibenclamide significantly decreased glucose AUC_0-4hr_ to the same extent at all doses, with AUC_0-4hr_ at 10 mg/kg reduced to 411.1 mg/dL*hr (Fig 4D).

### Effects of TMG-123 on glucose tolerance as an add-on to metformin

Metformin has been reported to reduce plasma glucose levels by reducing hepatic glucose production [23], and TMG-123 is assumed to improve the glucose tolerance by enhancing the hepatic glucose uptake. Since both TMG-123 and metformin may act on the same organ (i.e. liver), we evaluated the combined effects of TMG-123 and metformin. Administration of 300 mg/kg metformin in combination with 3 mg/kg TMG-123 significantly decreased the glucose AUC_0-4hr_ compared to individual administration of compounds (Fig 4E). This result shows that TMG-123 in combination with metformin exerts additional efficacy in ZDF rats.

### Effects of sub-chronic and chronic treatment with TMG-123 on HbA1c levels and plasma/liver TG in animal models of insulin-deficient and -resistant diabetes

**Fig 5:**
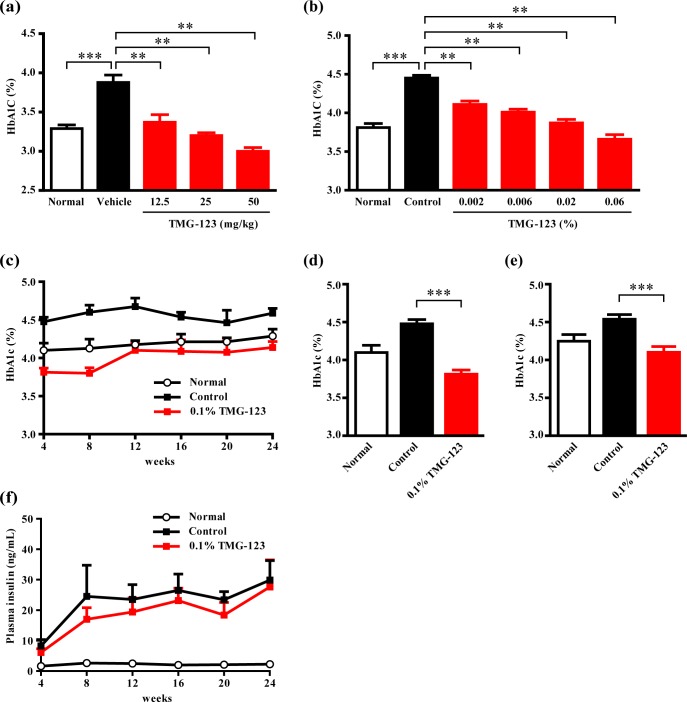
TMG-123 produces a sustained reduction in HbA1c levels in animal models of insulin-deficient and -resistant diabetes. (a) HbA1c levels in Goto-Kakizaki rats after once daily administration of TMG-123 per oral for 4 weeks (n = 9–10). (b) HbA1c levels in DIO mice after administration of TMG-123 as a diet admixture for 4 weeks (n = 10). (c-f) 24 weeks study in DIO mice (n = 8). (c) Time courses of HbA1c levels during a 24-week administration period of TMG-123. HbA1c levels at 4 (d) and 24 (e) weeks after initiating treatment. (f) Time courses of plasma insulin levels. **p < 0.025, ***p < 0.001.

To examine the durability of the glucose-lowering effect of TMG-123, we administered TMG-123 for 4 or 24 weeks and evaluated HbA1c levels in two animal models of T2DM. In an insulin-deficient diabetes animal model (Goto-Kakizaki rats), a 4-week oral administration of TMG-123 (12.5–50 mg/kg) significantly reduced HbA1c levels in a dose-dependent manner, with 50 mg/kg reducing HbA1c levels below those of the normal group (Wistar rats) (Fig 5A). In an insulin-resistant diabetes animal model (Diet-induced obesity (DIO) mice), 4-week dietary administration of TMG-123 (0.002–0.06%) significantly reduced HbA1c levels in a dose-dependent manner, with 0.06% reducing HbA1c levels below those of mice given a normal diet (Fig 5B). GK activators has been reported to exert favorable HbA1c reductions in patients with T2DM, while they also show attenuated effects in more than 8 weeks of treatment [11–13]. In the 24 weeks of administration study in DIO mice, HbA1c levels in the TMG-123-treated group were lower than those in control group throughout the administration period (Fig 5C), and differences between two groups were significant at 4 and 24 weeks (Fig 5D and 5E). These results suggest that TMG-123 reduces HbA1c levels in both insulin-deficient and -resistant diabetes models and that the efficacy of TMG-123 is sustained during chronic administration. In the 24-week study, TMG-123 did not increase plasma insulin levels compared to the control group (Fig 5F). This result suggests that TMG-123 reduces HbA1c levels without increasing plasma insulin levels, even after chronic administration.

**Fig 6:**
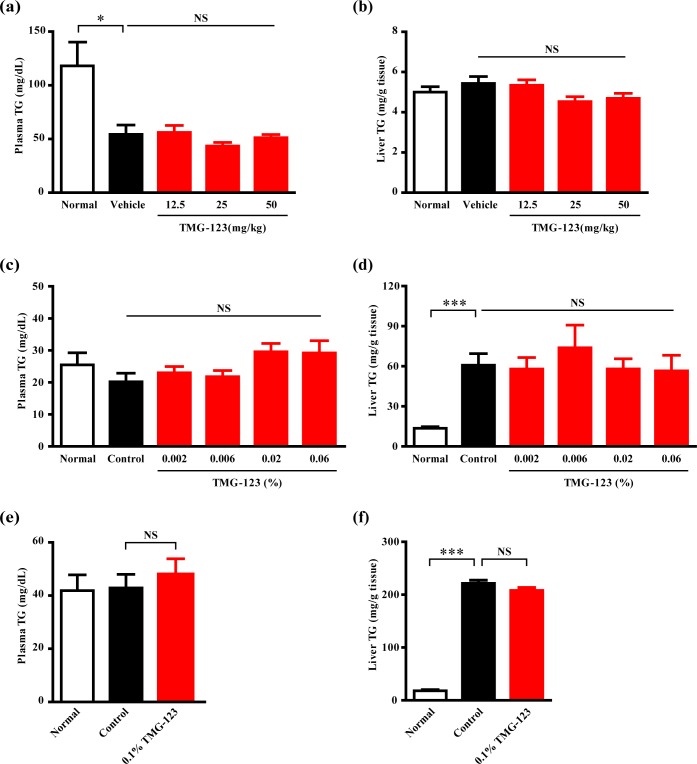
Sub-chronic and chronic administration of TMG-123 does not induced hypertriglyceridemia and liver TG accumulation in animal models of insulin-deficient and -resistant diabetes. (a, b) Plasma TG levels and liver TG contents after a 4-week administration in Goto-Kakizaki rats (n = 9–10). (c, d) Plasma and liver TG after a 4-week administration (n = 10) and (e, f) those after a 24-week administration (n = 8) in DIO mice. *p < 0.05, ***p < 0.001, NS = not significant.

It has been previously reported that more than treatment with GK activators for 12 weeks and over increases plasma TG levels in patients with T2DM [11, 12, 14]. In addition, previous preclinical studies have shown that other GK activators increase plasma TG in Goto-Kakizaki rats after 21 days of administration and liver TG in DIO mice after 4 weeks of administration [16, 24]. After 4 weeks of TMG-123 administration, plasma and liver TG did not increase compared with the control groups in Goto-Kakizaki rats (Fig 6A and 6B) and DIO mice (Fig 6C and 6D). Furthermore, even after 24 weeks of administration, the levels of plasma and liver TG were comparable to that of the control group in DIO mice (Fig 6E and 6F). In these studies, TMG-123 did not affect the body weights and food intakes (S2 Fig, S3 Fig). These results demonstrate that TMG-123 produces a sustained reduction in HbA1c levels without affecting plasma and liver TG in animal models of T2DM.

## Discussion

GK activators are expected to be novel antidiabetic drugs and several phase 2 clinical trials of other GK activators have demonstrated their significant glucose-lowering effects. However, these agents have also shown undesirable effects such as the loss of efficacy over time and an increase in plasma TG levels. GK activators need to overcome these concerns to become a new treatment option for T2DM. In this study, we examined the *in vitro* and *in vivo* pharmacological characteristics of a novel GK activator, TMG-123, and also evaluated the durability of efficacy and the effects on TGs following sub-chronic and chronic treatment. The *in vitro* study characterized the selective GK activation of TMG-123 without increasing Vmax. The *in vivo* studies demonstrated that TMG-123 exerted glucose-lowering effects without increasing insulin secretion in both insulin-deficient and -resistant models. Furthermore, a sustained reduction of HbA1c levels was observed without affecting hepatic and plasma TG, even after sub-chronic and chronic treatment. Thus, TMG-123 has glucose-lowering effect by mainly enhancing hepatic actions of GK and it is expected to be an antidiabetic drug that overcomes the concerns previously reported with other GK activators.

GK activators are divided into two types [25, 26]: those that lower blood glucose levels by activating GK systematically, which increases insulin secretion from pancreatic β-cells as well as hepatic glucose uptake, and those that lower blood glucose levels by activating GK hepatoselectively due to their high affinity for organic anion-transporting polypeptide family (OATP1B1, OATP1B3) expressed in liver. Since the present study showed that TMG-123 decreased plasma glucose levels without increasing plasma insulin levels in several animal models of T2DM (Figs 3, 4A and 4B), TMG-123 is likely to act primarily on the liver. Our preliminary study supports this characteristic of TMG-123; TMG-123 radioactivity was higher in liver than in pancreas after a single oral administration of [^3^H]TMG-123 to Sprague-Dawley rats (unpublished data). In contrast, since the cell-based assays in the present study indicated that EC_50_ value of TMG-123 in MIN6 cells was lower than that in rat primary hepatocytes (Fig 2), it appears to contradict the liver-dominant profile of TMG-123 mentioned above. In view of the enzyme assay results that indicated TMG-123 equally activates both liver and pancreas isoforms of GK (Table 1), it is possible that the sensitivities to GK activators intrinsically differed between primary hepatocytes and the β-cell line used in this study, which could be quite different from the *in vivo* circumstances. Further studies are needed to explore the mechanism underlying why TMG-123 does not stimulate insulin secretion *in vivo*.

As the loss of efficacy was noted in the clinical trials of two GK activators, MK-0941 and AZD1656, questions have arisen regarding the long-term efficacy of GK activators [11–13]. A previous preclinical report also showed that transgenic mice overexpressing GK developed impaired glucose tolerance over 6 months old, and the transgenic mice fed a high-fat diet became glucose intolerance and whole-body insulin resistance associated with enhanced weight gain [27]. In this study, we evaluated the durable efficacy of TMG-123 after 6 months administration in DIO mice. As a result, TMG-123 lowered HbA1c levels for 24 weeks of administration period (Fig 5C) and had no effects on the plasma insulin levels and the weight gain (Fig 5F, S2C Fig). The mechanism of the loss of efficacy has not been clarified, but it has been recently reported that pancreas-specific activation of GK causes hyperglycemia associated with dysfunction and death of β-cells in transgenic mice with an activating mutation [28]. Moreover, in rat primary islet culture, treatment with a GK activator in the presence of high glucose causes β-cell death [29]. From these data, we hypothesized that pancreatic β-cell dysfunction or GK activator-induced cell death may be at least partly responsible for the loss of efficacy. This study showed that the administration of TMG-123 did not alter plasma insulin levels compared with the control throughout 24 weeks of administration in DIO mice (Fig 5F), which is consistent with the results of OGTTs in Goto-Kakizaki rats, db/db mice, and ZDF rats. These results suggest the possibility that TMG-123 does not induce the β-cell dysfunction or death and could produce sustained efficacy.

With respect to the effects of GK activators on lipids, the increase in circulating lipids and lipid deposition in liver is a concern following a report that transgenic mice overexpressing GK exhibited high circulating lipids [27]. In fact, other GK activators increased hepatic and plasma TG after 4 days of treatment in db/db mice [17]. In addition, treatment with MK-0941 and AZD1656 increased plasma TG levels in patients with T2DM [11, 12]. The present study examined the effect of TMG-123 after chronic treatment in DIO mice. It has been previously reported that DIO mice show high lipid deposition in the liver [30], and our study also showed that hepatic TG is higher in DIO mice than mice receiving a normal diet (Fig 6D and 6F). In this animal model, TMG-123 did not affect hepatic and plasma TG after 24 weeks of administration (Fig 6E and 6F). Moreover, TMG-123 did not affect either TG levels after 4 weeks of administration in insulin-deficient Goto-Kakizaki rats (Fig 6A and 6B). These results suggest that TMG-123 may not affect lipid homeostasis. At present, the mechanism of hypertriglyceridemia caused by GK activation is not fully understood. A previous study demonstrated that transgenic mice, with GK activity reported to be about five-fold higher than that of controls, developed hypertriglyceridemia concomitant with the accumulation of hepatic TG with aging [27]. On the other hand, another study involving a more mild increase in hepatic GK activity (lower than two fold) did not show increased plasma TG levels [31] or hepatic TG content [32]. Another GK activator (MK-0941) elevated plasma TG after 14 weeks of treatment in a Phase 2 trial. It has been also reported that GK activation by MK-0941 was associated with increases in both glucose affinity and Vmax [33]. Therefore, it is possible that excess activation of hepatic GK over a long period could cause hypertriglyceridemia, while the ability of TMG-123 to activate GK without increasing Vmax (Fig 1) could be ideal for clinical use.

Judging from the clinical data previously reported, GK activators can be expected to exert comparable efficacy on HbA1c to other antidiabetic drugs, such as Sulfonylureas and DPP-4 inhibitors [12, 14, 15]. The present study demonstrated that TMG-123 improved glucose tolerance in both insulin-deficient and -resistant models, and the effects were greater than those of metformin and glibenclamide (Fig 4A, 4C and 4D). In addition, TMG-123 also normalized HbA1c levels in both insulin-deficient and -resistant models (Fig 5A and 5B). Since GK activation by TMG-123 does not differ among the species studied (human, rat, and mouse GK; Table 1), TMG-123 is expected to exert sufficient glucose-lowering effects in patients with T2DM. The American Diabetes Association and the European Association for the Study of Diabetes published a position statement on the management of hyperglycemia in patients with T2DM [2]. The statement recommends that mono-therapy should begin with metformin, with other antidiabetic drugs added as dual- or triple-therapy, providing that HbA1c does not fall below a target level [2]. Metformin lowers blood glucose levels by inhibiting hepatic gluconeogenesis, although its precise mechanisms of action remain unclear [23]. This study demonstrated that co-treatment with TMG-123 and metformin improved glucose tolerance compared with mono-therapy in ZDF rats (Fig 4E), although both TMG-123 and metformin were thought to act on the liver. The results indicate that the effects of TMG-123 and metformin do not interfere with each other. As is the case of PF-04991532, a hepatoselective GK activator, which lowered HbA1c levels in patients on stable doses of metformin [14], TMG-123 may produce an additional reduction in HbA1c levels in patients treated with metformin.

In this study, we examined the pharmacological characteristics of TMG-123 using animal models. We must be cautious of the facts that the pathogenesis of T2DM in animal models and human are not identical; we do not fully understand whether the contributions of GK to glucose homeostasis differ between humans and rodents and whether decreased HbA1c levels in animal models can reflect a beneficial effect on the prevention of microvascular and macrovascular complications in human. In addition, the underlying mechanisms of the risks of loss of efficacy and increased TG levels have not been elucidated in clinical trials. For these issues, clinical assessment of TMG-123 will be necessary to elucidate the pharmacological characteristics and risks. Since TMG-123 is undergoing Phase 1 studies, the clinical proof of concept for this compound will be clarified in near term.

In conclusion, this study demonstrated the pharmacological characteristics of TMG-123, and suggested that TMG-123 has glucose-lowering effects and reduces HbA1c levels during chronic treatment without affecting hepatic and plasma TG. In addition, TMG-123 can be utilized in combination therapy with metformin. Therefore, TMG-123 is a good candidate for the development of a new treatment in patients with T2DM.

## Supporting information

S1 Fig TMG-123 decreases S_0.5_ values of pancreas GK without increasing Vmax. Glucose concentration-versus-human pancreas GK activity relationships in the presence of 30 μM TMG-123 or vehicle alone (5% DMSO).
(PDF)

S2 Fig TMG-123 did not affect body weight. Body weight at (a) 4 week in 4-week study in Goto-Kakizaki rats (n = 9–10), (b) 4 week in 4-week study in DIO mice (n = 10), and (c) 24 week in 24-week study in DIO mice (n = 8). NS = not significant.
(PDF)

S3 Fig TMG-123 did not affect the amount of food intake. The average of daily food intake levels in (a) 4-week study in Goto-Kakizaki rats (n = 9–10) and (b) 4-week study in DIO mice (n = 10). (c) Daily food intake levels at 23 week in 24-week study in DIO mice (n = 8). NS = not significant.
(PDF)
