# Supplementary material for: TMG-123, a novel glucokinase activator, exerts durable effects on hyperglycemia without increasing triglyceride in diabetic animal models
Source: PLoS One. 2017 Feb 16;12(2):e0172252. doi: 10.1371/journal.pone.0172252 (PMC5313197; doi:10.1371/journal.pone.0172252)
Supplement: S1 Fig — Glucose concentration-versus-human pancreas GK activity relationships in the presence of 30 μM TMG-123 or vehicle alone (5% DMSO). (PDF) [file pone.0172252.s001.pdf]

Figure S1. TMG-123 decreases $S_{0.5}$ values of pancreas GK without increasing Vmax.

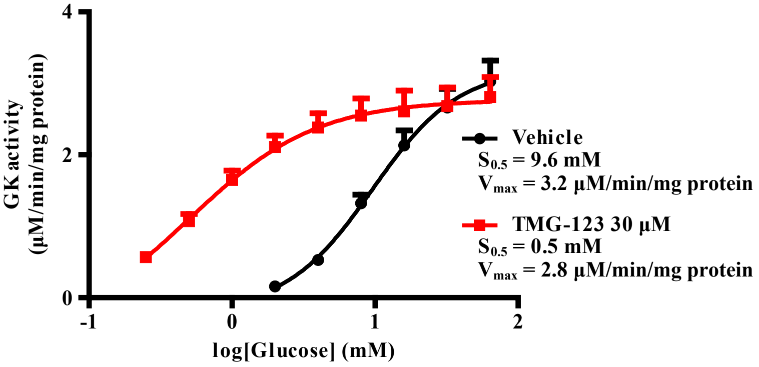

Glucose concentration-versus-human pancreas GK activity relationships in the presence of 30 µM TMG-123 or vehicle alone (5% DMSO).
