# Supplementary material for: TMG-123, a novel glucokinase activator, exerts durable effects on hyperglycemia without increasing triglyceride in diabetic animal models
Source: PLoS One. 2017 Feb 16;12(2):e0172252. doi: 10.1371/journal.pone.0172252 (PMC5313197; doi:10.1371/journal.pone.0172252)
Supplement: S2 Fig — Body weight at (a) 4 week in 4-week study in Goto-Kakizaki rats (n = 9–10), (b) 4 week in 4-week study in DIO mice (n = 10), and (c) 24 week in 24-week study in DIO mice (n = 8). NS = not significant. (PDF) [file pone.0172252.s002.pdf]

Figure S2. TMG-123 did not affect body weight.

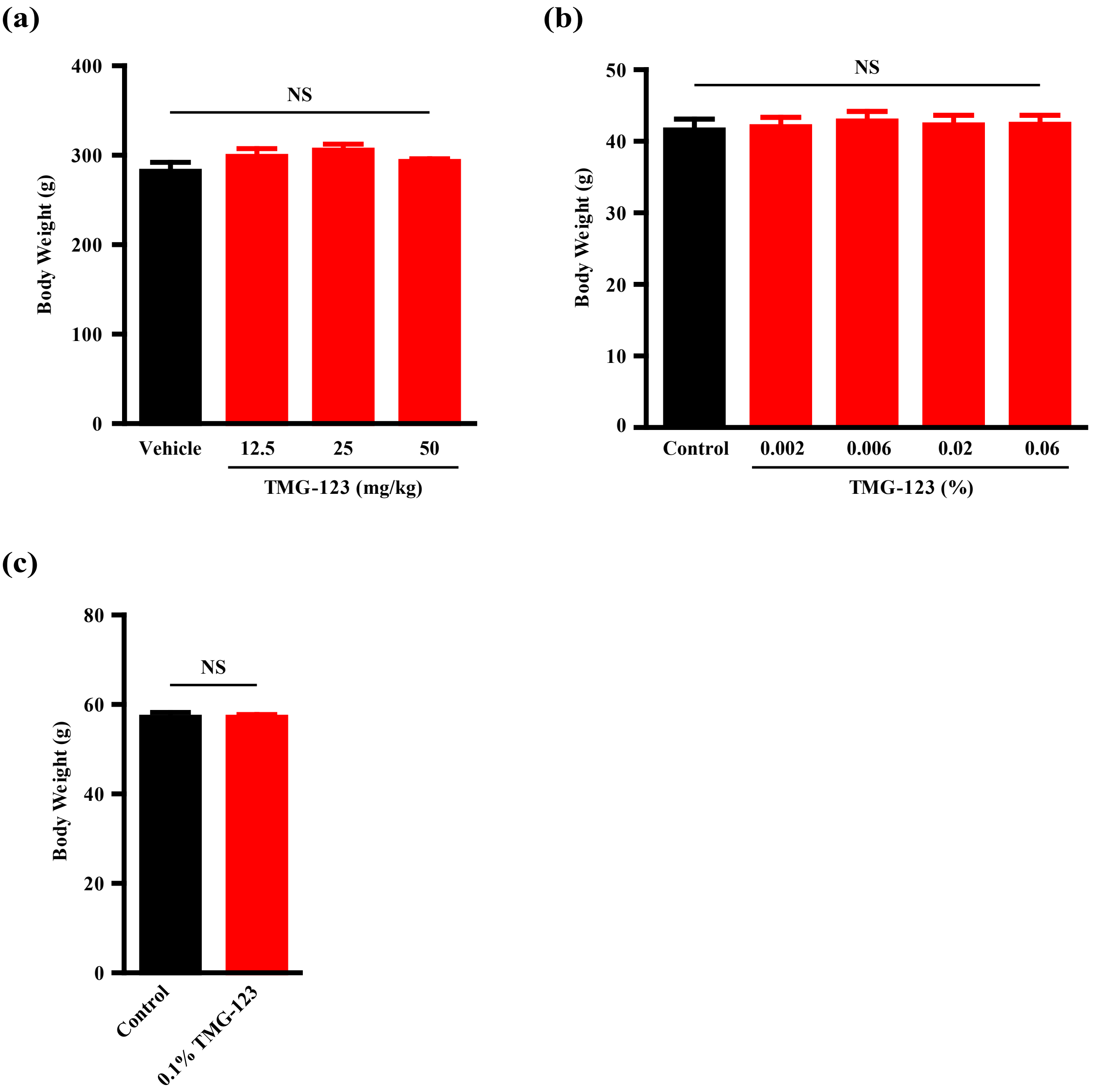

Body weight at (a) 4 week in 4-week study in Goto-Kakizaki rats (n=9-10), (b) 4 week in 4-week study in DIO mice (n=10), and (c) 24 week in 24-week study in DIO mice (n=8). NS = not significant.
