# Supplementary material for: TMG-123, a novel glucokinase activator, exerts durable effects on hyperglycemia without increasing triglyceride in diabetic animal models
Source: PLoS One. 2017 Feb 16;12(2):e0172252. doi: 10.1371/journal.pone.0172252 (PMC5313197; doi:10.1371/journal.pone.0172252)
Supplement: S3 Fig — The average of daily food intake levels in (a) 4-week study in Goto-Kakizaki rats (n = 9–10) and (b) 4-week study in DIO mice (n = 10). (c) Daily food intake levels at 23 week in 24-week study in DIO mice (n = 8). NS = not significant. (PDF) [file pone.0172252.s003.pdf]

Figure S3. TMG-123 did not affect the amount of food intake.

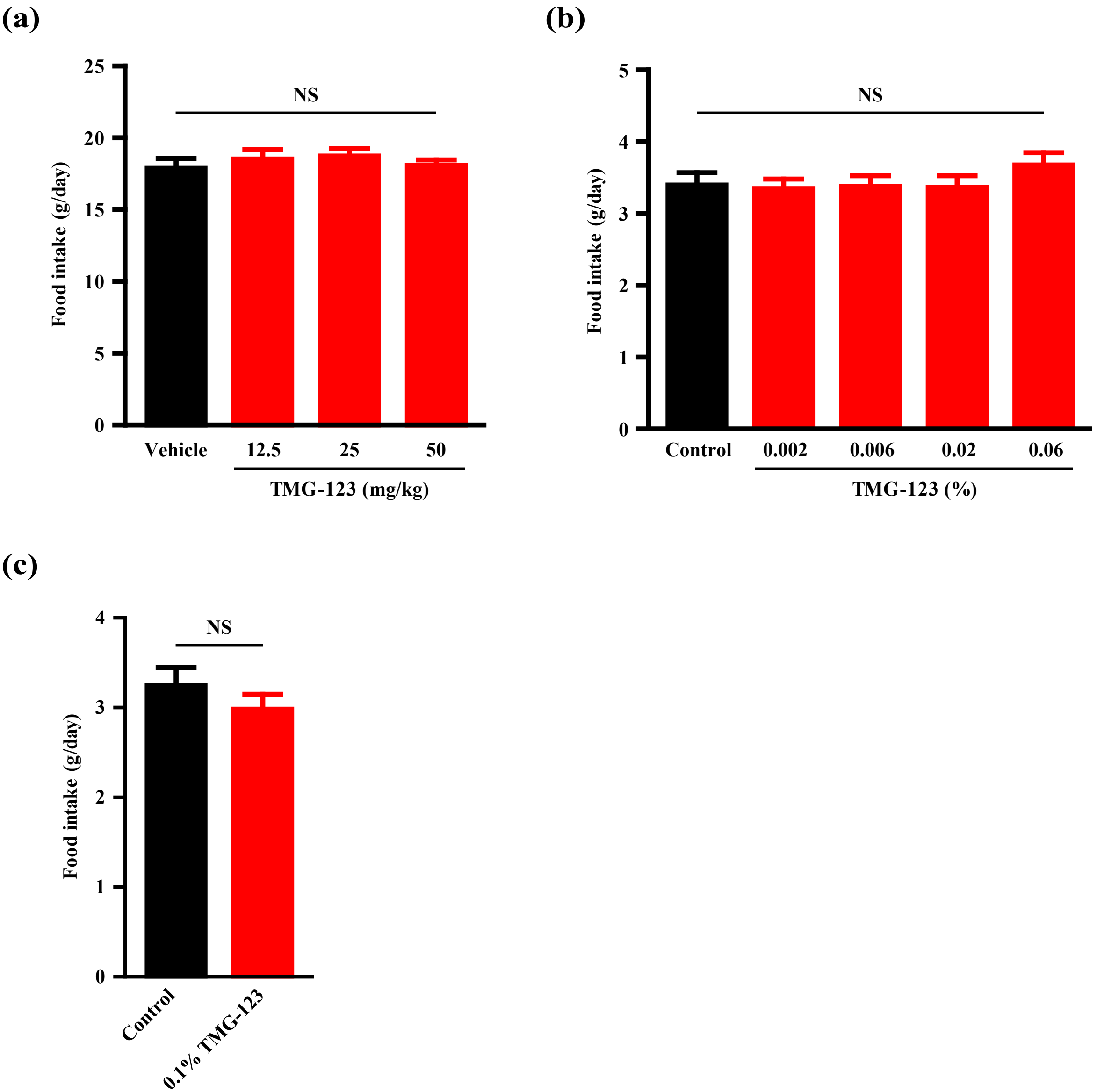

The average of daily food intake levels in (a) 4-week study in Goto-Kakizaki rats (n=9-10) and (b) 4-week study in DIO mice (n=10). (c) Daily food intake levels at 23 week in 24-week study in DIO mice (n=8). NS = not significant.
